# Impacts of adult illness on employment outcomes of rural households in India

**DOI:** 10.7189/jogh.08.020408

**Published:** 2018-12

**Authors:** Khurshid Alam, Andre Renzaho, Ajay Mahal

**Affiliations:** 1 School of Population and Global Health, The University of Western Australia, Perth, Australia; 2 Humanitarian and Development Research Initiative, School of Social Sciences and Psychology, Western Sydney University, Sydney, Australia; 3 Department of Epidemiology and Preventive Medicine, Monash University, Melbourne, Australia; 4 Nossal Institute for Global Health, The University of Melbourne, Melbourne, Australia

## Abstract

**Background:**

Existing literature on the impacts of adult illness on household labour supply and income in low- and middle-income countries shows that adverse health conditions significantly affect household labour supply, work participation and earnings. Most of the studies, however, are not equipped to distinguish between short- and long-term consequences of adult illness. We measured the impact of adult illness on household employment outcomes both in the short- and the long-run, using a unique longitudinal data set from rural India.

**Methods:**

We used two waves of India Human Development Survey (1993-94 and 2004-05) with a balanced panel of 10 726 households to assess short-run (in the year of the occurrence of adult illness) and long-run (after 11 years of the occurrence of adult illness) effects of major illness of adult household members aged 15-64 years on household employment outcomes, using multiple matching methods: nearest-neighbor matching and inverse probability weighting following propensity score matching, and coarsened exact matching to compare employment outcomes to a set of matched control households.

**Results:**

Rural households affected by adult illness experienced declines in workforce participation rate by 1-3%, wage employment by 4-15 days, and wage-earnings by Indian Rupee (INR) 374 to INR 837 compared to the matched control households in the short-run. In response, adult non-sick members of the affected households increased their workforce participation sharply by 14-16% to compensate for shortfalls in the short-run. In the long-run, workforce participation rate of the affected households also declined by nearly 1-3%. The long-run declines in wage-days and wage-earnings were small and not always statistically significant across the methods. However, long-run workforce participation rate of non-sick adults were smaller (4-6%) compared to short-run, but still statistically significant.

**Conclusions:**

The long-term effects were smaller in absolute magnitude than those of the short-run. This suggests coping and adjustments by the affected households using this 11-year longer time-span in a manner that helps to ameliorate the immediate impacts of adult illness. Our study also reiterates the importance of improving financial access to health services as well as access to social security benefits for the illness-affected households in rural India both in the short- and long-run.

There is a vast literature on the impacts of adult illness on household labour supply and income in low- and middle-income countries (LMICs). Studies have estimated the impacts of adult illness using both cross-sectional and longitudinal household surveys. Analyses based on cross-sectional data and short (eg, 1-year) panels show that adverse health conditions significantly affect household labour supply, work participation and earnings, although the magnitudes of the effects vary greatly across studies [1-8]. Studies using longitudinal data sets from Indonesia and China have demonstrated that illness of household head measured by worsening of health status significantly affect labour force participation, labour supply and labour earnings of the household [9,10]. Studies in Russia and Taiwan have shown that chronic conditions also lower labour-force participation and earnings [11,12]; and in Vietnam, the presence of a bedridden adult in the household reduced annual household work-days by 8% [13].

The above findings on adverse earnings and employment effects of illness are not surprising given the heavy reliance of household members on informal employment in LMICs, with little access to social protection in the form of medical coverage, unemployment insurance and job protection. It is likely that a household member with a disease will experience lower work participation, with the household potentially experiencing increased work and labour force participation of (other) non-sick members to compensate for any financial shortfalls [9,14,15]. In this context, a consideration of longer-term impacts can be important because a longer time-span would allow adjustments by households that ameliorate the immediate impacts of adult illness. For example, some households (or their members) may migrate to locations that offer better employment opportunities [16]. Intra-household labour substitution may also be possible with household members, with the potentially higher earners in the labour market (usually adult males) able to increasingly replace earnings by those with low net returns to labour, such as women. Although not explored in this paper, improved opportunities for inter-temporal adjustments within households may also lead later-born cohorts to be disproportionately allocated to schooling (or away from labour) by households experiencing an adult illness. Of course, lowered permanent incomes on account of adult illness will have the opposite effect.

Most of the existing studies, however, are not equipped to distinguish between short- and long-term consequences of adult illness, although a recent study from Chile showed that a severe illness (defined as a hospital stay by a household member) lowered employment and earnings both in the short-run (current year of illness) and 4 years after the original illness [17]. The authors showed that one-year effects were larger in absolute magnitude than 4-year effects, and that as the length of hospital stay increased, the adverse earning effects became larger.

In this paper we focus on the impact of adult illness on household employment outcomes both in short-run (in the year of the occurrence of adult illness) and long-run (after 11 years of the occurrence of adult illness) using a unique longitudinal data set from India. This data set includes a rich set of variables on employment in India, including workforce participation, days spent working for wages, income from wages, cropped area and irrigated cropped area. Quite apart from our contribution to the international literature on the longer-term implications of adult illness, our paper also adds to the very limited literature that exists on the economic implications of adult illness in India, all of it focused on short-term impacts. These studies in India using cross-sectional surveys showed adult-illness reduce work participation and incomes of adult household members at different magnitude [3,18-20].

## METHODS

### Matching

We used three matching methods: i) nearest-neighbor matching and ii) inverse probability weighting (IPW) [21] following propensity score matching (PSM) [22], and iii) coarsened exact matching (CEM) [23], to compare labour supply and earning outcomes in the short-run (in the 12 months preceding the 1993-94 survey) and after 11 years (in the 12 months preceding 2004-05 survey) for households that experienced adult illness (treatment households) in the 12 months preceding the 1993-94 survey to a set of matched control households that did not.

The PSM procedure involves two steps [24]. In the first step, the probability (the “propensity score”) that a household is affected by adult illness, was predicted based on observed household characteristics (“pre-treatment” covariates). This (pre-processing) step involves estimating a logit model with household socioeconomic, demographic and locational characteristics. The second step involves matching treatment households to control households with similar propensity scores using a matching algorithm. In this paper, we used the nearest-neighbour matching algorithm.

Balance checking of pre-treatment covariates is typically a key step in PSM methods [23]. We compared the means of covariates used in the logit model of treatment and control households using the so-called “standardized bias” – the differences in means between treated and matched control households divided by the square-root of the average of the sample variances of the two groups – with the requirement that this measure be less than 25% [25]. In spite of the above mentioned balance checks, there is always the risk that PSM might lead to the inclusion of treatment and control households with very different socioeconomic and demographic characteristics when using a summary measure such as the propensity scores.

CEM is an alternative matching approach where households with adult illness and control households are exactly matched, but after ‘coarsening’ of the variables [26]. For example, instead of exact age (in years), a coarsening entails specifying age-categories to which a respondent belongs – such as 0-14 years, 15-29 years, and so on. CEM does not entail checking for covariates balancing unlike matching based on propensity scores, as households are matched exactly on each (coarsened) covariate and not on the propensity scores. Although this approach retains larger sample size for matching than matching exactly on all household characteristics, sample attrition can still be significant and remains a limitation of this method. And conditional independence is assumed by CEM.

Another method that lowers the risk of imbalance is IPW. As in the case of PSM, the first step in using IPW is the generation of propensity scores. The IPW method uses weights (based on the inverse of the propensity score) to create a synthetic sample such that the distribution of measured baseline covariates is independent of treatment assignment. Larger weights are assigned to “underrepresented” observations, and lower weight to “overrepresented” observations as indicated by propensity scores. A weighted regression (with the inverse of the propensity scores as weights) was used to generate estimates of the impact of adult illness under the IPW method.

### Robustness checks

Matching methods such as PSM are nonparametric and typically require a common support restriction involving dropping treatment observations whose p score is higher than the maximum or less than the minimum pscore of the controls. The quality of the matches may thus be improved, but the cost is loss of observations, making PSM methods sensitive to common support. So, we explored the implication of the loss of observations due to imposing common support restrictions for our results. Relatedly, the thinness of overlap in propensity scores may also be a concern. To assess the resulting implications for employment outcomes, we estimated the impact of further restricting the common support region by dropping treatment and control households with the lowest density (in the respective empirical distributions). In particular, we dropped between 1% to 10% of the households with the lowest density for propensity scores to assess the sensitivity of our results to assumptions about common support [27].

Second, estimates from matching methods cannot be interpreted causally unless the so-called “conditional independence assumption” (CIA) is satisfied. In our case, CIA implies that conditional on the observed covariates using for matching, the distribution of treatment households is statistically independent of the potential household economic outcomes (that is, outcomes in the absence of adult illness). Unfortunately, it is not possible to directly test the validity of this assumption; so, we adopted a strategy suggested in the literature to evaluate the robustness of our economic impact estimates based on PSM to violations of CIA. The strategy to assess the implications of a violation of the CIA requires assuming that CIA does not hold and the existence of a convenient unobserved binary variable (say U), so that if U were observed and included in the set of matching variables, CIA would, indeed, be satisfied [28]. Alternative assumptions on the distribution of U determine the likelihood of selection into adult illness-affected households, the magnitude of potential economic outcomes of interest (whether above or below the sample mean) in the absence of adult illness and the impact estimates if U were observed and used to generate propensity scores for matching. In our sensitivity analysis, we explored how large the selection and outcome effects had to be to overturn our findings on the labor supply and earnings effects of adult illness on households [28,29]. Technically, we first assessed the impact of U on our findings of economic impacts under different hypothetical scenarios, with each scenario reflecting (1) the odds of selection into adult illness-affected household when the binary variable U = 1, vs the odds of selection when U = 0 and (2) the odds of potential outcomes taking a value greater than the sample mean when U = 1 vs the odds of potential outcomes taking value greater than the sample mean when U = 0, in the adult illness-affected household. We also examined the implications of including an unobservable with the same distribution as an already existing binary variable in our sample—namely, whether the affected household experienced adult illness in the second period (2004-05). At present, no comparable set of methods do exist for CEM to address conditional independence.

Third, we tried to address “variable selection” by dropping less relevant co-variates in propensity score that drive our treatment variable (adult illness). We assessed changes in our outcome estimates if the number of covariates are reduced. We did this under two additional scenarios – checking the robustness of our findings to a reduced number of covariates.

Finally, we applied the following fixed effect (FE) regression in panel data analysis to examine current employment effects of adult illness (current illness) using a 2-wave panel data (1993-94, 2004-2005):


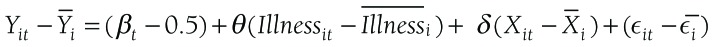


Where,

*Y_it_ – Ῡ_i_* change in current employment outcomes

*Illness_it_ – Illness_i_*acute illness

*X_it_ – X_i_* dummy covariates

*ϵ_it_ – ϵ_i_* error term

θ FE coefficient

Using FE we looked at changes over time over the same household, all we examined was “within” (individual) variation. That is, the coefficient θ we estimated is primarily based on the covariance (correlation) between *Y_it_ – Ῡ_i_* and *Illness_it_ – Illness_i_*. Thus we estimated the economic impacts of major illness in the current period (not the long-term impact of illness). As we are taking deviations from the mean, we are (in effect) also ruling out illnesses that show up for the individual household in both periods, that is chronic illnesses (because they are part of the mean). Thus, we ended up with is the impact of acute illnesses.

The main advantage of the FE method is that it removes bias introduced by heterogeneous individual characteristics. However, it is still possible that other sources of endogeneity remain. For example, illness in the previous period may affect economic outcomes in the previous period, and these economic outcomes in the past period may influence future economic opportunities.

So, we estimated long-term employment effect of the lagged illness in following equation:


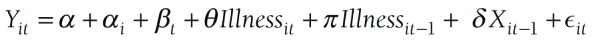


Where,

*Y_it_*long-term employment outcomes (wave 2)

*Illness_it–1_* lagged illness (wave 1)

*X_it–1_* all dummy covariates (wave 1)

*Illness_it_*major illness (wave 2)

*ϵ_it_* error term

*π* regression coefficient

This specification is well known to have endogeneity problems (apart of the standard heterogeneity across households). However, it is likely that the long time period between rounds (11 years) may have helped flush out “most” of the endogeneity. With this specification, and with only 2 rounds of data, this is essentially a regression model where we only used 2004-2005 outcomes (wave 2) and the lagged illness variable from 1993-1994 (wave 1) and all the X variables from 1993-1994 (wave 1).

### Data

We used data from the Human Development Profile of India (HDPI) Survey and the India Human Development Survey (IHDS), both carried out by the National Council for Applied Economic Research (NCAER). The HDPI survey was implemented in 1993-94 and covered 33 230 rural households in 1765 villages and 195 districts across 16 states in India. The HDPI used a stratified three stage sampling design and its sampling frame included more than 95% of India’s rural population. The HDPI survey collected information on household and individual level socio-demographic and economic characteristics, such as age, sex, religion, whether scheduled caste or tribe, income and land ownership. The survey also collected detailed information on employment, earnings, health care utilization, indicators of health and educational status, and some categories of household expenditures.

The IHDS was carried out in 2004-2005 as a follow-up to the HDPI and is a nationally representative, survey of 41 554 households in 1503 villages and 971 urban neighbourhoods located in 276 towns and cities across India. About 65% of the sample consisted of rural households, and IHDS collected data on household expenditures and incomes, along with household- and individual-level data on education, heath, employment, marriage, fertility, indicators of social capital, landownership, farming practices and crop diversity. In states where HDPI was conducted, re-contact details were available for 13 593 households for a follow-up in the 2004-2005 IHDS. The IHDS interviewed 10 791 households from HDPI as well as an additional 2290 households in 2004-2005, some of whose members were part of the set of 10 791 households in 1993-94. In effect, information was available for 13 081 households in 2004-2005, consisting of members of the initial set of 10 791 households. Hence, we constructed a balanced panel data set of 10 791 households combining HDPI (1993-94) and IHDS (2004-05). However, after merging IHDS with HDPI, only 10 726 households could be matched in this manner. For both HDPI and IHDS, the initial ethics approval was obtained from the Ethics Review Committee of the NCAER, New Delhi and the Institutional Review Board of the University of Maryland, College Park, USA. For this study, the Monash University Human Research Ethics Committee granted exemption from further ethical review (reference No.CF12/1442-2012000778).

### Treatment variable

We constructed a dummy of adult illness as the treatment variable, with the dummy taking the value 1 if a household experienced a major illness (epilepsy, hypertension, diabetes, heart disease, mental illness, tuberculosis, leprosy, cancer) of a member aged 15-64 years in the year preceding the 1993-94 HDPI survey.

### Matching variables

To account for observable confounders in the relationship between adult illness and employment outcomes, we used the following household characteristics from 1993-94 survey to construct propensity scores (for the PSM method) and for matching households under the CEM method:

#### Household characteristics

These included socio-demographic characteristics, living conditions and characteristics of the head of the household.

Socio-demographic characteristics included were: (a) a dummy equal to 1 if household size was less than the average rural household size based on the Census of India 2001 (equal to 5.6), 0 otherwise; (b) dummy for cultivable land ownership (1 if the household owned land, 0 otherwise); (c) 15 state dummies (1 if the household belonged to a specific state, 0 otherwise); (d) a dummy for major religion (1 if the household is Hindu, 0 otherwise); and (e) a dummy for caste or tribal status (1 if the household belonged to a scheduled caste or tribe, 0 otherwise). Indicators of living conditions were: (a) type of house (1 if the house was kutcha, 0 otherwise); (b) the number of rooms in the house (1 if 3 or more rooms, 0 otherwise); (c) separate kitchen in the house (1 if the house had a separate kitchen, 0 otherwise); (d) type of cooking stove (1 if the cooking stove was smoke emitting, 0 otherwise); (e) electricity supply (1 if the house had an electricity connection, 0 otherwise); (f) toilet (1 if there was any toilet within the household, 0 otherwise); (g) improved toilets (1 if the household had toilet facilities with septic tank or sealed water pits, 0 otherwise) and; (h) improved drinking water sources (1 if the household had access to water from the protected well, tanker truck, pipe outside or inside the house or hand pipe, 0 otherwise) [30].

Characteristics of the household head that were included: (a) age (1 if the age of household head was 15-64 years, 0 otherwise); (b) sex (1 if male, 0 otherwise); and (c) indicator of educational attainment (1 if completed at least secondary education, 0 otherwise).

### Employment outcomes

#### Absence from work-days in last one year per adult

We constructed a measure of days absent from work per adult by dividing the total number of days absent from work by the number of all working age adult members (15-64 years) of the household.

#### Adult workforce participation rate in last one year

Adult individuals aged 15-64 years were considered to be working if they were engaged in one or more gainful activities during the year preceding the two surveys – whether working on household farms or businesses, or for wages or salaries. We constructed household-level adult workforce participation rate by dividing the total number of adult workers by the number of all working age adult members (15-64 years) of the household. Similarly, we constructed a household-level adult workforce participation rate for non-sick adults aged 15-64 years.

#### Wage-days per adult in last one year

The number of days spent working for wages per adult (15-64 years) in the previous year was constructed by dividing the total wage-days by the number of adults of the household to assess household employment other than participating in work on the family farm.

#### Wage-income per adult in last one year

We constructed a measure of household wage-income per adult by dividing all wage-incomes by the number of adult (15-64 years) household members [31]. We inflated 1993-94 wage-income data using the World Bank’s consumer price index for India to make it comparable with 2004-05 wage-income.

#### Per capita cropped and irrigated cropped area in last one year

A measure of per capita cropped area [32] in bigha (local unit) in the last one year was constructed by dividing total cropped area of a household by household size. We further constructed a measure of per capita irrigated cropped area [32, 33] in bigha (local unit) in last one year, dividing total irrigated cropped area of a household by household size.

### Subgroup analysis

We examined employment outcomes of adult illness-affected households by subgroups of socioeconomic status, particularly (a) scheduled caste and tribe (SC/ST), two groups that are considered historically deprived in India, vs non-SC/ST households, and (b) households having cultivable land, vs households without cultivable land. To assess group-specific differences in employment outcomes, we estimated linear probability models, using ordinary least square (OLS) regressions on a data set consisting only of matched households based on nearest-neighbour matching and CEM. For IPW, we run OLS regression on the unmatched data (with propensity scores) but using the inverse of the propensity scores as the sampling weight. The outcomes were the Y variable, with the X variables being an indicator for whether a household was affected by adult illness, an indicator for the specific sub-groups of interest (eg, SC/ST, cultivable land ownership) and the product (interaction) of the indicators for the household with adult illness and sub-group. The coefficient of the interaction term was used to assess sub-group differences.

## RESULTS

The summary statistics of treatment households, unmatched control households and matched control households with nearest-neighbour matching (using propensity scores) are presented in **Table 1**. The comparison between households affected by adult illness and controls show that the means of indicators for the socioeconomic, demographic and locational characteristics of matched households are considerably closer to the corresponding means for households affected by adult illness, relative to unmatched controls (the matches between treatment and control households would, of course, be even closer if we used CEM). Estimates of “standardized bias” are reported in the last column of **Table 1** and are less than 10% in all cases, considerably much less than the 25% threshold recommended in Ho et al. in 2007 [25]. The data show that 97% of the treatment households are headed by males, 85% households are Hindu and the 69% households own at least some cultivable land.

**Table 1:** Summary statistics of the matching variables by treated, matched control and unmatched control households

Matching variable	Treated households (95% CI)	Control households-matched (95% CI)	Control households-unmatched (95% CI)	% bias
State1 dummy (%)	7.02‡ (6.07 - 7.97)	5.63‡ (4.93 - 6.33)	5.96 (5.43 - 6.49)	5.71
State2 dummy (%)	8.06 (7.05 - 9.07)	7.45 (6.65 - 8.25)	6.73 (6.16 - 7.30)	2.28
State3 dummy (%)	2.21 (1.67 - 2.75)	2.10 (1.67 - 2.53)	6.83 (6.26 - 7.40)	0.76
State4 dummy (%)	7.63 (6.65 - 8.61)	7.78 (6.97 - 8.59)	7.65 (7.05 - 8.25)	-0.56
State5 dummy (%)	8.31 (7.29 - 9.33)	8.27 (7.43 - 9.11)	4.40 (3.94 - 4.86)	0.15
State6 dummy (%)	2.10 (1.57 - 2.63)	1.96 (1.54 - 2.34)	2.32 (1.98 - 2.66)	0.99
State7 dummy (%)	8.56 (7.52 - 9.60)	8.66 (7.81 - 9.51)	11.74 (11.01 - 12.47)	-0.36
State8 dummy (%)	18.54† (17.10 - 19.98)	20.39† (19.17 - 21.61)	13.76 (12.98 - 14.54)	-4.67
State9 dummy (%)	7.13 (6.18 - 8.08)	7.27 (6.48 - 8.06)	7.40 (6.81 - 7.99)	-0.54
State10 dummy (%)	6.45 (5.54 - 7.36)	6.02 (5.30 - 6.74)	5.66 (5.14 - 6.18)	1.78
State11 dummy (%)	6.99 (6.05 - 7.93)	7.49 (6.69 - 8.29)	9.43 (8.77 - 10.09)	-1.93
State12 dummy (%)	4.21 (3.47 - 4.95)	3.92 (3.33 - 4.51)	5.73 (5.20 - 6.26)	1.47
State13 dummy (%)	3.46 (2.78 - 4.14)	3.89 (3.30 - 4.48)	6.60 (6.04 - 7.16)	-2.29
State14 dummy (%)	9.27 (8.20 - 10.34)	9.13 (8.26 - 10.00)	5.65 (5.13 - 6.17)	0.48
State15 dummy (%)	0.07 (-0.03 - 0.17)	0.04 (-0.02 - 0.10)	0.13 (0.05 - 0.21)	1.28
Household head: male (%)	96.58 (95.91 - 97.25)	97.15 (96.65 - 97.65)	95.28 (94.80 - 95.76)	-3.27
Household head: adult (15-64 years) (%)	90.48 (89.39 - 91.57	91.69 (90.85 - 92.53)	88.70 (87.98 - 89.42)	-4.25
Household head: high school completion (%)	34.83 (33.07 - 36.59)	32.80 (31.38 - 34.22)	35.10 (34.02 - 36.18)	4.29
Religion-Hindu (%)	84.78 (83.45 - 86.11)	85.92 (84.87 - 86.97)	86.26 (85.48 - 87.04)	-3.22
Schedule tribe & schedule cast (%)	35.33 (33.56 - 37.10)	35.37 (33.92 - 36.82)	35.40 (34.32 - 36.48)	-0.08
Cultivable land ownership (%)	69.30 (67.59 - 71.01)	71.27 (69.90 - 72.64)	66.10 (65.03 - 67.17)	-4.31
Household size ≤ 5.6 (%)	43.96 (42.12 - 45.80)	43.17 (41.67 - 44.67)	50.67 (49.54 - 51.80)	1.59
House type-kutcha (not brick-built) (%)	52.19 (50.34 - 54.04)	53.98 (52.47 - 55.49)	50.45 (49.32 - 51.58)	-3.59
House with 3 rooms and more (%)	38.57 (36.77 - 40.37)	38.40 (36.93 - 39.87)	31.34 (30.29 - 32.39)	0.35
House with separate kitchen (%)	48.09 (46.24 - 49.94)	47.99 (46.48 - 49.50)	43.56 (42.44 - 44.68)	0.20
Smoke emitting stove (%)	93.76† (92.86 - 94.66)	94.83† (94.16 - 95.50)	93.21 (92.64 - 93.78)	-4.61
Household with electricity (%)	53.69 (51.84 - 55.54)	51.84 (50.32 - 53.36)	51.93 (50.80 - 53.06)	3.71
Toilet within household (%)	13.08§ (11.83 - 14.33)	10.05§ (9.14 - 10.96)	11.25 (10.54 - 11.96)	9.49
Improved toilet (%)	10.05§ (8.94 - 11.16)	7.95§ (7.13 - 8.77)	8.61 (7.98 - 9.24)	7.34
Improved water sources (%)	67.38 (65.65 - 69.11)	67.91 (66.49 - 69.33)	71.79 (70.78 - 72.81)	-1.13
Sample	2805	4178	7528	–

CI – confidence interval, y – years

*Estimates are means from the Human Development Profile of India 1993-94. In columns (1)-(3) 95% confidence intervals are reported in parentheses below the proportions. For matching purposes, propensity score calculations are based on logit regression estimates. The standardized bias (% Bias) reported in column (4) refers to the difference of the sample means of the adult illness-affected and control households as a percentage of the square root of the average of the sample variances in the adult illness-affected and matched control households. Statistically significant difference between the treatment and matched controls households are shown at the significance level of 1%, 5%, and 10% based on the nearest-neighbour algorithm.

†*P* < 0.10.

‡*P* < 0.05.

§*P* < 0.001.

**Table 2** reports the results of the first-stage logit regressions for generating propensity scores for a household being affected by adult illness. Nearly half of the coefficients are statistically indistinguishable from zero. However, the characteristics of the household head, household size, and indicators of housing conditions such as households having 3 and more rooms, households having improved toilets and having access to improved water sources are significantly associated with being affected by adult illness.

**Table 2:** Estimates of logit regression models for stage 1 of propensity score matching for the adult illness-affected households in rural India*

Matching variable	Regression coefficient (SE)
State1 dummy (Yes = 1, No = 0)	1.02 (0.79)
State2 dummy (Yes = 1, No = 0)	1.02 (0.78)
State3 dummy (Yes = 1, No = 0)	-0.26 (0.79)
State4 dummy (Yes = 1, No = 0)	0.83 (0.79)
State5 dummy (Yes = 1, No = 0)	1.36 † (0.79)
State6 dummy (Yes = 1, No = 0)	0.64 (0.79)
State7 dummy (Yes = 1, No = 0)	0.56 (0.79)
State8 dummy (Yes = 1, No = 0)	1.10 (0.78)
State9 dummy (Yes = 1, No = 0)	0.76 (0.78)
State10 dummy (Yes = 1, No = 0)	0.92 (0.79)
State11 dummy (Yes = 1, No = 0)	0.46 (0.79)
State12 dummy (Yes = 1, No = 0)	0.60 (0.79)
State13 dummy (Yes = 1, No = 0)	0.14 (0.79)
State14 dummy (Yes = 1, No = 0)	1.32 † (0.78)
Household head-male dummy (Yes = 1, No = 0)	0.33§ (0.12)
Adult household head (15-64 years) dummy (Yes = 1, No = 0)	0.33§ (0.08)
Household head-high school completion dummy (Yes = 1, No = 0)	-0.12‡ (0.05)
Religion-Hindu dummy (Yes = 1, No = 0)	-0.09 (0.07)
Schedule tribe/ schedule cast dummy (Yes = 1, No = 0)	0.04 (0.05)
Cultivable land ownership dummy (Yes = 1, No = 0)	0.07 (0.05)
Household size ≤ 5.6 dummy (Yes = 1, No = 0)	-0.21§ (0.05)
House type-kutcha (not brick-built) dummy (Yes = 1, No = 0)	0.02 (0.05)
House with 3 rooms and more dummy (Yes = 1, No = 0)	0.18§ (0.06)
House with separate kitchen dummy (Yes = 1, No = 0)	0.09† (0.05)
Smoke emitting stove dummy (Yes = 1, No = 0)	0.07 (0.10)
Household with electricity dummy (Yes = 1, No = 0)	0.10† (0.06)
Toilet within household dummy (Yes = 1, No = 0)	0.24† (0.14)
Improved toilet dummy (Yes = 1, No = 0)	-0.09 (0.16)
Improved water sources dummy (Yes = 1, No = 0)	-0.17§ (0.05)
Constant	-2.39§ (0.81)
Number of observations	10,333
Pseudo R2	0.03

*Estimates are based on estimates from a logit regression model used to generate propensity scores, using data from the Human Development Profile of India 1993-94. Standard errors are reported in parentheses below the coefficient estimates; Statistical significance are shown at 1%, 5%, and 10%.

†*P* < 0.10.

‡*P* < 0.05.

§*P* < 0.001.

**Figure 1:**
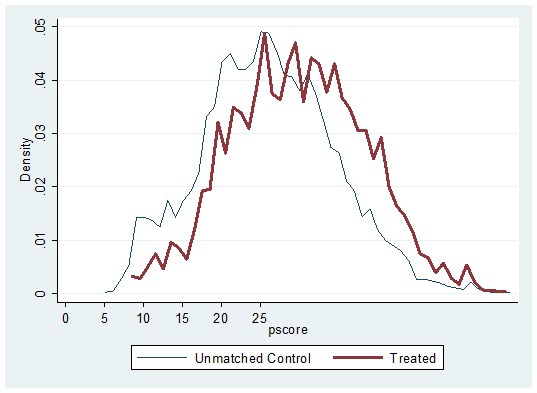
Distribution of propensity scores for adult illness-affected households and unmatched controls.

**Figure 1** shows the empirical distribution of propensity scores for adult illness-affected households and their respective unmatched controls. In general, the empirical distributions of adult illness-affected households and control households track each other well, so we could expect non-trivial matches over the region of common support. The support for unmatched controls fully contains the support for households affected by adult illness, so the standard common support restriction did not lead to any loss of observation in the treatment group. However, the implications for cases where treatment and control households had a low density for propensity scores are further explored in sensitivity analyses, the results for which are reported in Table SA1 in **Online Supplementary Document(Online Supplementary Document)**.

Adult illness increased household out-of-pocket (OOP) health expenditure per adult (15-64 years) for the treatment households by 835-997 Indian Rupee (INR) in the year they occurred but the effect was smaller (98-115 INR) after 11 years of adult illness compared to matched controls. Analogously, hospitalized days for the treatment households increased by 5 days per adult (15-64 years) in the year of adult illness but after 11 years hospitalized days per adult in treatment households were statistically indistinguishable from that for matched controls (these results are not presented in the table). These results show that major illness is associated with increased hospitalization and household OOP spending in the short-run.

**Table 3** presents estimates of short-run and 11-year effects on employment outcomes for rural households in India affected by adult illness. Although not all of the results are statistically distinguishable from zero, the results for nearest-neighbour matching and IPW following PSM, and CEM methods are generally consistent with each other and allow a few broad conclusions. Absence from work-days per adult increased by seven days relative to matched controls in the short-run and after 11 years the effects were much smaller (increased by one day). The adult (15-64 years) workforce participation rate in treatment households decreased by nearly 1-3% relative to matched controls (overall participation rate was 65% for the treated vs 68% for control households following nearest-neighbour matching) in the short-run, depending on the matching method used. The workforce participation rate after 11 years of adult illness also declined among the affected households in similar magnitude by nearly 1-3%. Similarly, days spent in wage-based work (“wage-days”) per adult aged 15-64 years in treatment households decreased by 4-15 days (117 for the treated vs 121 days for control households following nearest-neighbour matching) in the short-run, but the 11-year effects were small and not always statistically significant across the methods. Wage income (in the last one year) per adult also declined by INR 374 to INR 837 in the short-run depending on the matching methods used. After 11-year wage earnings per adult also continued to decline but these declines were not statistically distinguishable from zero.

**Table 3:** Short-term and long-term employment effects on Indian rural households affected by adult illness: results from nearest-neighbour matching, inverse probability weighting and coarsened exact matching*

Outcome variable	Nearest-neighbour matching		Inverse probability weighting		Coarsened exact matching	
1993-94	2004-05	1993-94	2004-05	1993-94	2004-05
Absent from work-days per adult (15-64 y) in last one year	6.76§ (0.23)	1.30‡ (0.60)	6.70§ (0.25)	1.44§ (0.57)	6.99§ (0.21)	0.84 (0.59)
Workforce participation among adults (15-64 y) in last one year (%)	-2.59§ (0.75)	-2.53§ (0.78)	-2.79§ (0.65)	-2.33§ (0.67)	-1.19 (0.74)	-0.71 (0.79)
Workforce participation of non-sick adults (15-64 y) in last one year (%)	14.54§ (0.72)	4.37§ (0.77)	14.27§ (0.61)	4.61§ (0.66)	15.84§ (0.72)	6.17§ (0.78)
Wage-days per adult (15-64 y) of household in last one year	-3.93 (2.58)	-2.55 (1.96)	-15.14§ (3.16)	-3.55‡ (1.74)	-10.45§ (3.28)	-2.32 (1.95)
Wage-income per adult (15-64 y) in last one year (INR)	-373.51§ (103.93)	-181.34 (118.76)	-836.99§ (119.46)	-140.69 (106.71)	-628.50§ (132.38)	-122.38 (112.92)
Per capita cropped area by household in last one year	-0.87‡ (0.38)	0.11 (0.32)	-0.63‡ (0.31)	-0.06 (0.29)	-0.46 (0.39)	-0.10 (0.25)
Per capita irrigated cropped area by household in last one year	-0.68§ (0.28)	0.02 (0.18)	-0.19 (0.20)	0.10 (0.21)	-0.40 (0.26)	-0.13 (0.22)
Sample = Treatment + Control	2805 (4178)	2805 (4178)	10277	10072	1840 (3443)	1840 (3443)

y – years

*Coefficients are the average treatment effect applying nearest-neighbour matching, inverse probability weighting and coarsened exact matching using the Human Development Profile of India 1993-94 and India Human Development Survey 2004-05. Standard errors are reported in parentheses below coefficient estimates. Income and expenditure data in Indian Rupees in 1993-94 were inflated using the World Bank’s consumer price index for India to make them comparable with 2004-05.For each coefficient, statistical significant differences between the treatment and matched controls were shown at the level of 1%, 5%, and 10%.

†*P* < 0.10.

‡*P* < 0.05.

§*P* < 0.001.

Our results also show that work participation rates of non-sick adults (15-64 years) rose sharply in the short-run by 14-16% (83% for the treated vs 68% for the matched control households under nearest-neighbour matching). After11 years, these effects were smaller (4-6%), but still statistically significant. **Table 3** also shows a decline in per capita cropped area by 0.5-0.9 bighas (local unit) and per capita irrigated cropped area by 0.2-0.7 bighas (local unit) among households affected by adult illness in the short-run. However, in the longer run (11 years after the adult illness), the changes in per capita cropped area and per capita irrigated cropped area were smaller in magnitude and often statistically insignificant.

Our subgroup results by SC/ST status are presented in **Table 4**. The subgroup analysis shows that in the short-run the SC/ST households experienced a greater number of work-days missed per adult compared to non-SC/ST households. The SC/ST households also experienced higher loss in wage-income per adult compared to their non-SC/ST counterparts in the short-run. These findings are stable across the three estimation methods we employed.

**Table 4:** Short-term and long-term employment effects on Indian rural households affected by adult illness: Results by schedule caste/schedule tribe (SC/ST) status*

Outcome variable	Nearest-neighbour matching (1993-94)		Nearest-neighbour matching (2004-05)		Inverse probability weighting (1993-94)		Inverse probability weighting (2004-05)		Coarsened exact matching (1993-94)		Coarsened exact matching (2004-05)	
SC/ST	Non-SC/ST	SC/ST	Non-SC/ST	SC/ST	Non-SC/ST	SC/ST	Non-SC/ST	SC/ST	Non-SC/ST	SC/ST	Non-SC/ST
Absent from work-days per adult (15-64 y) in last one year	7.35§ (0.29)	6.44§ (0.22)	0.98 (0.81)	1.68§ (0.61)	7.32§ (0.44)	6.36§ (0.30)	0.94 (0.82)	1.71‡ (0.75)	7.44§ (0.31)	6.68§ (0.24)	1.01 (0.88)	1.47‡ (0.67)
Workforce participation among adults (15-64 y) in last one year (%)	-3.00§ (1.03)	-4.08§ (0.77)	-2.19‡ (1.09)	-2.80§ (0.82)	-2.37‡ (1.05)	-2.96§ (0.80)	-2.06† (1.11)	-2.43§ (0.84)	-1.56 (1.13)	-1.31 (0.87)	-1.67 (1.20)	-0.57 (0.92)
Workforce participation of non-sick adults (15-64 y) in last one year (%)	13.95§ (1.01)	13.16§ (0.76)	4.44§ (1.09)	4.24§ (0.82)	14.23§ (0.94)	14.36§ (0.78)	4.43§ (1.09)	4.75§ (0.83)	15.65§ (1.11)	15.31§ (0.85)	4.61§ (1.93)	6.65§ (0.91)
Wage-days per adult (15-64 y) of household in last one year	-20.42§ (4.00)	-14.56§ (3.96)	-3.41 (2.68)	-4.50‡ (2.03)	-19.11§ (4.53)	-11.17§ (4.40)	-3.49 (2.99)	-3.28 (2.08)	-15.98§ (4.31)	-6.26 (4.38)	-2.66 (2.94)	-3.41 (2.25)
Wage-income per adult (15-64 y) in last one year (INR)	-843.73§ (162.95)	-443.13§ (160.80)	-394.72‡ (162.41)	-79.14 (122.71)	-1042.30§ (165.00)	-639.60§ (171.34)	-271.34 (187.82)	-53.45 (126.16)	-880.05§ (175.69)	-267.37 (178.53)	-357.62‡ (176.72)	22.01 (135.07)
Per capita cropped area by household in last one year	-0.44 (0.48)	-0.65† (0.36)	0.66 (0.48)	-0.12 (0.32)	-0.11 (0.33)	-0.95‡ (0.44)	0.77 (0.66)	-0.25 0.30)	-0.29 (0.57)	-0.32 (0.44)	-0.27 (0.44)	-0.15 (0.29)
Per capita irrigated cropped area by household in last one year	0.08 (0.33)	-0.38 (0.25)	0.23 (0.40)	0.06 (0.24)	0.18 (0.21)	-0.42 (0.28)	0.50 (0.59)	-0.04 (0.20)	0.05 (0.39)	-0.47 (0.30)	0.03 (0.45)	-0.02 (0.27)
Sample = Treatment+Control	7504	7504	7362	7362	7504	7504	7362	7362	6224	6224	6113	6113

SC/ST – schedule caste/schedule tribe, y – years

*Coefficients are the average treatment effect applying inverse probability weighting and coarsened exact matching using the Human Development Profile of India 1993-94 and India Human Development Survey 2004-05 by schedule caste/schedule tribe status. Standard errors are reported in parentheses below coefficient estimates. For each coefficient, statistical significance are shown at the level of 1%, 5% and 10%. Income and expenditure data in Indian Rupees in 1993-94 were inflated using the World Bank’s consumer price index for India to make them comparable with 2004-05.

†*P* < 0.10.

‡*P* < 0.05.

§*P* < 0.001.

Our second subgroup results by cultivable land ownership status are presented in **Table 5**. The subgroup analysis shows that the effects on workforce participation rates among adults in the landless rural households were significantly lower compared to the households having cultivable land ownership in the short-run. On the other hand, both per capita cropped area and per capita cropped irrigated area decreased markedly more among households with cultivable land ownership compared to households that did not own land. Although statistical significance was not always achieved, these findings were generally consistent across the three methods we used.

**Table 5:** Short-term and long-term employment effects on Indian rural households affected by adult illness: Results by cultivable land ownership status*

Outcome variable	Nearest-neighbour matching (1993-94)		Nearest-neighbour matching (2004-05)		Inverse probability weighting (1993-94)		Inverse probability weighting (2004-05)		Coarsened exact matching (1993-94)		Coarsened exact matching (2004-05)	
**Land**	**Non-land**	**Land**	**Non-land**	**Land**	**Non-land**	**Land**	**Non-land**	**Land**	**Non-land**	**Land**	**Non-land**
Absent from work-days per adult (15-64 y) in last one year	6.63§ (0.21)	7.06§ (0.31)	1.14† (0.60)	1.38 (0.89)	6.59§ (0.28)	6.91§ (0.48)	1.62‡ (0.67)	1.18 (1.06)	6.86§ (0.23)	7.44§ (0.35)	1.02 (0.67)	0.06 (1.01)
Workforce participation among adults (15-64 y) in last one year (%)	-2.87§ (0.76)	-6.38§ (1.11)	-2.23§ (0.79)	-3.88§ (1.17)	-2.19§ (0.77)	-3.94§ (1.19)	-2.12§ (0.78)	-3.42§ (1.24)	-0.93 (0.84)	-2.90‡ (1.25)	-1.23 (0.88)	-2.20† (1.33)
Workforce participation of non-sick adults (15-64 y) in last one year (%)	13.84§ (0.74)	11.70§ (1.10)	3.85§ (0.79)	4.82§ (1.17)	14.62§ (0.72)	13.66§ (1.14)	4.01§ (0.77)	5.12§ (1.24)	15.48§ (0.82)	15.82§ (1.25)	4.83§ (0.88)	6.98§ (1.33)
Wage-days per adult (15-64 y) of household in last one year	-18.96§ (3.69)	-13.88§ (4.24)	-3.40† (1.94)	-6.51‡ (2.87)	-21.44§ (3.70)	-5.10 (5.20)	-2.33 (1.86)	-2.58 (3.37)	-8.52‡ (4.08)	-6.83 (4.74)	-3.12 (2.14)	-0.05 (3.24)
Wage-income per adult (15-64 y) in last one year (INR)	-567.51§ (144.73)	-611.78§ (166.66)	-220.63‡ (117.06)	-142.46 (173.27)	-844.36§ (129.74)	-616.98§ (197.58)	-184.03† (96.26)	-130.70 (242.23)	-544.65§ (160.45)	-567.25§ (186.44)	-195.35 (126.14)	12.61 (190.65)
Per capita cropped area by household in last one year	-0.97§ (0.33)	-0.04 (0.49)	0.30 (0.28)	0.12 (0.67)	-1.41§ (0.42)	-0.03 (0.02)	0.13 (0.30)	-0.34 (0.84)	-0.93‡ (0.40)	-0.04 (0.61)	0.02 (0.27)	-0.83 (0.65)
Per capita irrigated cropped area by household in last one year	-0.60‡ (0.25)	-0.02 (0.36)	0.24 (0.21)	0.15 (0.49)	-0.50† (0.28)	-0.02‡ (0.01)	0.19 (0.24)	-0.29 (0.40)	-0.83§ (0.29)	-0.01 (0.44)	0.16 (0.25)	-0.12 (0.59)
Sample = Treatment + Control	7470	7470	7315	7315	7470	7470	7315	7315	6228	6228	6102	6102

y – years

*Coefficients are the average treatment effect applying inverse probability weighting and coarsened exact matching using the Human Development Profile of India 1993-94 and India Human Development Survey 2004-05 by cultivable land ownership status. Standard errors are reported in parentheses below coefficient estimates. For each coefficient, statistical significance are shown at the level of 1%, 5%, and 10%.

†*P* < 0.10.

‡*P* < 0.05.

§*P* < 0.001.

### Sensitivity analyses

We re-estimated household employment and earnings impacts under alternative “trimming” assumptions, ranging from dropping 1% to 10% of total treatment households that had propensity scores with a low density in the empirical distribution. The results of this analysis, which are summarized in Appendix Table A1 in **Online Supplementary Document(Online Supplementary Document)** show that trimming of the sample of adult illness-affected households with low density propensity scores does not influence our findings on the magnitude of the estimated impacts or the overall conclusions in the PSM analysis.

Although we cannot directly test for the CIA, our simulation results for the nearest-neighbour PSM (in Table SA2 in **Online Supplementary Document(Online Supplementary Document)**) suggest that an unobserved confounder with a distribution similar to that of U (mimicking small to large selection and outcome effects) does not overturn our main findings. The smaller the selection and outcome effects associated with U, the closer the simulated average treatment effect on the treated (ATT) estimates to our base ATT estimates reported in **Table 3**. For outcomes associated with negative ATT such as adult workforce participation, wage-days per adult and wage-income per adult, the larger the selection and outcome effects of the simulated confounder the ATT estimates become even larger in absolute magnitude (larger negatives). When ATT effects are positive (eg, absent from work-days, non-sick adult workforce participation) we can choose distributions of U that drive ATT estimates towards zero. However, the sizes of the selection effects required to overturn our results are rather ‘large’. Moreover, including a confounder with a distribution similar to that of adult illness in 2004-05 does not much alter our findings on long-term impacts, with the exception of wage incomes per adult. We conclude that our key results are fairly robust to violations of the CIA of the type assessed in this article, at least for results based on the PSM approach.

Our robustness check for “variable selection” under two additional scenarios –our findings to a reduced number of covariates (from 30 to 25 and 22 covariates, respectively) did not fundamentally change our original results on the employment impacts of adult illness. Dropping co-variates have just increased our total matched control sample sizes, but sample of matched treatment remained same. The results are presented in Tables SA3 and SA4 in **Online Supplementary Document(Online Supplementary Document)**, respectively.

Finally, applying FE regression in panel data analysis to examine current employment effects of adult illness and long-term employment effects of lagged illness using regression also did not change our original results from matching methods. The results are presented in the column 2 and 3, respectively, of Table SA5 in **Online Supplementary Document(Online Supplementary Document)**.

## DISCUSSION

We present evidence that rural Indian households, when faced with an adult illness, experience negative impacts on labour supply and wage-incomes in the short-run. These include a lowering of adult workforce participation rate, wage-days per adult and a lower wage-incomes from work. Our results also show that the affected households try to compensate for the declines in labour supply and incomes in the short-run by increasing work participation of non-sick adult household members. Our findings are consistent with previous literature. For example, Gertler and Gruber showed that in Indonesia, in response to the illness of head of households, the labour supply of other family members signiﬁcantly increased [9]. Berloffa and Modena found that poor Indonesian households increased labour supply by remaining family members to compensate for income losses in the face of sickness and death [14]. Similarly, Yamauchi et al. reported that in South Africa labour force participation increased by 20% among adolescents and adult women in response to income shocks due to adult deaths [15]. Despite the increase in work participation of the existing non-sick household adult members, per adult wage-earnings declined significantly in the short-run among households affected by adult illness, compared to their matched counterparts. This decline in wage-earning is unsurprising, given the lack of standard social security systems in rural India to compensate loss in household earnings due to illness. Reasons for the observed decline in per capita cropped area and per capita irrigated area in households affected by adult illness in the short-run is possibly include both the loss of household workers due to disability and the need to increase wage-income from outside work to pay for increased health expenses.

Our sub-group analyses show socioeconomically deprived groups such as households belonging to SC/ST experience a greater loss in wage-days and wage-incomes than their non-SC/ST counterparts. We also find that landless households that are more dependent on selling labour services tend to experience a larger decline in workforce participation because of adult illness compared to the households with land ownership. Analogously, the households with land ownership experienced consistently higher declines in both per capita cropped area and per capita irrigated cropped area.

We cannot entirely reject the hypothesis that adult illness has no long-run effects on household employment and earning outcomes in rural India. It is true that in most cases, we find the magnitude of the employment and earnings effects to be much lower in the long-run than in the short-run and sometimes the results are statistically insignificant. This suggests coping and adjustments by the affected households using this 11-year longer time-span in a manner that helps to ameliorate the short-term impacts of adult illness. This may be through informal support systems at the community level and households’ own responses to mitigate the adverse economic impacts of adult illness in the long-run. For example, if the households have access to micro credit, or sources of informal credit, they may be able to better protect their non-medical consumption and future economic vulnerability [34,35]. Intra-household adjustment in the form of better division of labour between wage work, agricultural work and household work could also become more efficient over time [36,37]. Overall though, adult illness continues to exert a significant burden on affected households by decreasing household wage incomes in the long-run.

There are limitations to our analysis. In the construction of the treatment variable, we excluded child illness assuming child illness has no direct impact on household labour supply. However, child illness requires care and support from the adult household members and this can negatively influence adult employment outcomes as well. Neglecting child illness can contribute to underestimation of the impacts of household employment outcomes. Our analysis also does not control for adult illness that households may have experienced over an 11-year period because the survey did not collect any information on major illnesses that may have occurred in the interim. While our sensitivity analyses sought to address confounding due to such events, it is possible (given the distributional assumptions we could impose on U) that our estimates do not adequately address this concern. We also could not adequately investigate the factors driving long-term impacts, as we lacked data on informal and formal credit markets and support systems in the survey. Nonetheless, our examination of “variable selection” and application of alternative methods such as FE regression in panel data analysis and regression of lagged illness on long-term employment outcomes confirm our original findings from the three matching methods.

Our analysis points to the importance of improving financial access to health services. Specifically, our work suggests that major health expenses and possible loss of a breadwinner can lead to intra-household adjustments in labour supply and income losses, some of which may persist even over a decade. Increased workforce participation by non-sick members, particularly by children may have adverse implications for human capital formation in the household such as educational attainment, and this is partially reflected in some of our findings on increase in workforce participation among children. It is also likely that efforts to fund health care expenses arising from adult illness may be inefficient, both because members whose skills are less remunerative may be forced to participate in the workforce. The risk of income losses following an adult illness also depends on access to social security benefits such as income protection against disability and sickness, unemployment benefits. These are frequently missing in LMICs such as India, except the small minority involved in formal sector work and primarily in urban areas. Thus a second policy implication of our work is the need to focus on social security for rural and other informal sector workers in LMICs.

## CONCLUSIONS

We conclude that the rural households remarkably decrease their labour supply and incomes in the short-run as a result of adult illness. The affected households also loss their labour supply and incomes in the long-run. But the long-term impacts of adult-illness on households were smaller in absolute magnitude than those of the short-run. This indicates coping and adjustments by the affected households using longer time-span after the incidence of adult-illness. Our study suggests improving financial access to health services as well as financial risk protection from major illness for the affected households. We also emphasize the importance of access to social security benefits for the illness-affected households. Finally, we call to activate effective health and social policies in rural India so that no household suffers undue financial hardship as a result of illnesses both in the short- and long-run.
