# Supplementary material for: Impacts of adult illness on employment outcomes of rural households in India
Source: J Glob Health. 2018 Jul 29;8(2):020408. doi: 10.7189/jogh.08.020408 (PMC6083015; doi:10.7189/jogh.08.020408)
Supplement: Online Supplementary Document [file jogh-08-020408-s001.pdf]

Online Supplementary Document

Alam et al. Impacts of adult illness on employment outcomes of rural households in India

J Glob Health 2018;8:020408

Table SA1: Estimates of average treatment effect on the treated (ATT) for employment outcomes for adult illness-affected households applying nearest-neighbour matching under alternative assumptions about common support

Employment outcomes	Trimming cut-off	ATT	Standard Error	Treated (N)	Controls (N)
Absent from work-days per adult (15-64 years) in last one year					
1993-94	Baseline	6.76***	0.23	2,805	7,472
	Trim (1)	6.76***	0.23	2,777	7,472
	Trim (2.5)	6.78***	0.23	2,735	7,472
	Trim (5)	6.82***	0.24	2,665	7,472
	Trim (10)	6.86***	0.24	2,527	7,472
2004-05	Baseline	1.18**	0.58	2,714	7,358
	Trim (1)	1.12**	0.58	2,687	7,358
	Trim (2.5)	1.06*	0.59	2,649	7,358
	Trim (5)	1.11*	0.60	2,580	7,358
	Trim (10)	1.13*	0.62	2,443	7,358

Workforce participation among adults (15-64 years) in last one year (%)					
1993-94	Baseline	-3.02***	0.69	2,805	7,472
	Trim (1)	-2.68***	0.70	2,777	7,472
	Trim (2.5)	-3.09***	0.71	2,735	7,472
	Trim (5)	-2.66***	0.72	2,665	7,472
	Trim (10)	-3.19***	0.74	2,527	7,472
2004-05	Baseline	-2.26***	0.74	2,714	7,358
	Trim (1)	-2.65***	0.75	2,687	7,358
	Trim (2.5)	-2.24***	0.76	2,649	7,358
	Trim (5)	-2.30***	0.77	2,580	7,358
	Trim (10)	-2.49***	0.79	2,443	7,358
Workforce participation of non-sick adults (15-64 years) in last one year (%)					
1993-94	Baseline	14.28***	0.68	2,698	7,472
	Trim (1)	14.38***	0.68	2,672	7,472
	Trim (2.5)	14.50***	0.69	2,632	7,472
	Trim (5)	15.21***	0.70	2,565	7,472
	Trim (10)	14.67***	0.72	2,430	7,472
2004-05	Baseline	4.29***	0.74	2,653	7,358
	Trim (1)	4.34***	0.74	2,627	7,358

	Trim (2.5)	4.43***	0.75	2,589	7,358
	Trim (5)	4.43***	0.76	2,522	7,358
	Trim (10)	4.50***	0.79	2,389	7,358
Wage-days per adult (15-64 years) of household in last one year					
1993-94	Baseline	-9.48***	3.16	1,247	3,429
	Trim (1)	-9.72***	3.19	1,235	3,429
	Trim (2.5)	-9.62***	3.22	1,216	3,429
	Trim (5)	-10.47***	3.29	1,185	3,429
	Trim (10)	-8.75***	3.35	1,124	3,429
2004-05	Baseline	-2.28	1.83	2,714	7,358
	Trim (1)	-1.49	1.83	2,687	7,358
	Trim (2.5)	-2.13	1.86	2,649	7,358
	Trim (5)	-1.13	1.88	2,580	7,358
	Trim (10)	-2.35	1.95	2,443	7,358
Wage-income per adult (15-64 years) in last one year (INR)					
1993-94	Baseline	-563.20***	126.70	1,247	3,429
	Trim (1)	-563.23***	127.80	1,235	3,429
	Trim (2.5)	-567.16***	127.85	1,216	3,429
	Trim (5)	-509.85***	128.63	1,185	3,429
	Trim (10)	-595.94***	134.04	1,124	3,429
2004-05	Baseline	-80.34	112.32	2,714	7,358

	Trim (1)	-112.21	111.19	2,687	7,358
	Trim (2.5)	-59.58	112.85	2,649	7,358
	Trim (5)	-149.98	114.96	2,580	7,358
	Trim (10)	-19.99	116.44	2,443	7,358
Per capita cropped area by household in last one year					
1993-94	Baseline	-0.70**	0.35	2,805	7,528
	Trim (1)	-0.86**	0.36	2,777	7,528
	Trim (2.5)	-0.70*	0.37	2,735	7,528
	Trim (5)	-0.95***	0.38	2,665	7,528
	Trim (10)	-0.98***	0.38	2,527	7,528
2004-05	Baseline	-0.24	0.36	1,909	4,921
	Trim (1)	-0.27	0.37	1,891	4,921
	Trim (2.5)	-0.47	0.41	1,865	4,921
	Trim (5)	-0.25	0.38	1,814	4,921
	Trim (10)	-0.52	0.45	1,719	4,921
Per capita irrigated cropped area by household in last one year					
1993-94	Baseline	-0.62**	0.27	2,805	7,528
	Trim (1)	-0.50*	0.26	2,777	7,528
	Trim (2.5)	-0.47*	0.26	2,735	7,528
	Trim (5)	-0.68**	0.28	2,665	7,528
	Trim (10)	-0.66**	0.28	2,527	7,528

2004-05	Baseline	-0.01	0.25	1,088	2,864
	Trim (1)	0.01	0.26	1,078	2,864
	Trim (2.5)	-0.02	0.26	1,061	2,864
	Trim (5)	-0.05	0.27	1,034	2,864
	Trim (10)	-0.18	0.28	983	2,864

Note: Trim imposes common support by dropping **x** percent of the treatment observations at which the density of the empirical distribution of propensity scores is the lowest. Statistical significance are shown at 1%***, at 5%**, and at 10%*. Income and eexpenditure data in Indian Rupees in 1993-94 were inflated using the World Bank's consumer price index for India to make them comparable with 2004-05.

Table SA2: Sensitivity analysis for employment effects of adult illness on household in rural India following nearest-neighbour matching: average treatment effect on the treated (ATT)

**Outcome variable**	**Short-run employment effect (1993-94)**			**Long-run employment effect (2004-05)**		
	**ATT (SE)**	**Outcome effect**	**Selection effect**	**ATT (SE)**	**Outcome effect**	**Selection effect**
Absent from work-days per adult (15-64 years) in last one year: Baseline result	6.76*** (0.23)	-	-	1.30** (0.60)	-	-
Adult illness (15-64 years) in 2004-05	-	-	-	1.11*** (0.38)	35.47	1.21

Simulation p11(0.8), p10(0.8), p01(0.6), p00(0.3)	6.76*** (<0.01)	4.57	9.41	-0.82* (0.45)	3.55	7.83
Simulation p11(0.8), p10(0.8), p01(0.55), p00(0.25)	6.76*** (<0.01)	4.57	12.12	-1.21** (0.59)	3.66	9.83
Simulation p11(0.8), p10(0.8), p01(0.5), p00(0.2)	6.76*** (<0.01)	5.70	16.16	-1.95*** (0.64)	4.04	12.69
Workforce participation among adults (15-64 years) in last one year (%): Baseline result	-2.59*** (0.75)	-	-	-2.53*** (0.78)	-	-
Adult illness in 2004-05	-	-	-	-1.91*** (0.45)	0.74	1.21
Simulation p11(0.8), p10(0.8), p01(0.6), p00(0.3)	-7.34*** (0.56)	3.51	5.43	-6.13*** (0.53)	3.55	4.61
Simulation p11(0.8), p10(0.8), p01(0.55), p00(0.25)	-8.17*** (0.57)	3.67	6.69	-6.82*** (0.59)	3.70	5.65
Simulation p11(0.8), p10(0.8), p01(0.5), p00(0.2)	-9.35*** (0.65)	4.04	8.40	-7.79*** (0.60)	4.08	6.98
Workforce participation of non-sick adults (15-64 years) in last one year (%): Baseline result	14.54*** (0.72)	-	-	4.37*** (0.77)	-	-
Adult illness in 2004-05	-	-	-	5.00*** (0.49)	0.68	1.18

Simulation p11(0.8), p10(0.8), p01(0.6), p00(0.3)	9.82*** (0.53)	3.53	5.43	0.63 (0.57)	3.51	5.05
Simulation p11(0.8), p10(0.8), p01(0.55), p00(0.25)	8.96*** (0.56)	3.67	6.72	-0.25 (0.54)	3.70	6.19
Simulation p11(0.8), p10(0.8), p01(0.5), p00(0.2)	7.81*** (0.57)	4.04	8.35	-1.24*** (0.59)	4.09	7.58
Wage-days per adult (15-64 years) of household in last one year: Baseline result	-3.93 (2.58)	-	-	2.55 (1.96)	-	-
Adult illness in 2004-05	-	-	-	-1.99* (1.15)	0.65	1.19
Simulation p11(0.8), p10(0.8), p01(0.6), p00(0.3)	-19.23*** (2.21)	3.54	3.71	-12.42*** (1.40)	3.53	5.19
Simulation p11(0.8), p10(0.8), p01(0.55), p00(0.25)	-21.11*** (2.47)	3.68	4.53	-14.35*** (1.52)	3.68	6.45
Simulation p11(0.8), p10(0.8), p01(0.5), p00(0.2)	-24.14*** (2.37)	4.04	5.65	-16.73*** (1.48)	4.03	7.97
Wage-income per adult (15-64 years) in last one year (INR): Baseline result	-373.51*** (103.93)	-	-	-181.34 (118.76)	-	-
Adult illness in 2004-05	-	-	-	-78.65 (62.07)	0.65	1.20

Simulation p11(0.8), p10(0.8), p01(0.6), p00(0.3)	-923.56*** (89.43)	3.53	3.68	-676.05*** (77.31)	3.53	5.85
Simulation p11(0.8), p10(0.8), p01(0.55), p00(0.25)	-1001.85*** (97.95)	3.71	4.51	-786.88*** (91.37)	3.69	7.20
Simulation p11(0.8), p10(0.8), p01(0.5), p00(0.2)	-1130.00*** (104.81)	4.04	5.56	-937.08*** (97.71)	4.06	9.01
Per capita cropped area by household in last one year: Baseline result	-0.87** (0.38)	-	-	0.11 (0.32)	-	-
Adult illness in 2004-05	-	-	-	0.16 (0.14)	0.95	1.20
Simulation p11(0.8), p10(0.8), p01(0.6), p00(0.3)	-1.83*** (0.31)	3.51	6.46	-0.43* (0.25)	3.51	5.34
Simulation p11(0.8), p10(0.8), p01(0.55), p00(0.25)	-1.96*** (0.31)	3.74	8.08	-0.60** (0.29)	3.69	6.60
Simulation p11(0.8), p10(0.8), p01(0.5), p00(0.2)	-2.28*** (0.38)	4.06	10.20	-0.74*** (0.30)	4.05	8.30
Per capita irrigated cropped area by household in last one year: Baseline result	-0.68*** (0.28)	-	-	0.02 (0.18)	-	-
Adult illness in 2004-05	-	-	-	0.06 (0.10)	0.87	1.22
Simulation p11(0.8), p10(0.8), p01(0.6), p00(0.3)	-1.30*** (0.24)	3.55	6.86	-0.28* (0.17)	3.53	3.99

Simulation p11(0.8), p10(0.8), p01(0.55), p00(0.25)	-1.41*** (0.23)	3.72	8.45	-0.39** (0.19)	3.72	4.78
Simulation p11(0.8), p10(0.8), p01(0.5), p00(0.2)	-1.68*** (0.28)	4.01	10.87	-0.46** (0.19)	4.00	5.90

Note: The probability of having confounder (U) =1 if treatment (T) =1 and outcome (Y) =1 (above the mean) is p11; The probability of *U* =1 when T=1 and Y=0 is p10; the probability of *U* =1 when T=0 and Y=1 is p01; and the probability of *U* =1, when T=0 and Y=0 is p00. This defines the probability distribution of the confounder. ***significant at 1% **significant at 5%; *significant at 10%. Income and expenditure data in Indian Rupees in 1993-94 were inflated using the World Bank's consumer price index for India to make them comparable with 2004-05.

Table SA3: Short-term and long-term employment effects on Indian rural households affected by adult illness: results from nearest-neighbour matching, inverse probability weighting and coarsened exact matching (5 co-variates dropped: sex of household head, religion, household size, dummy of household with three or more rooms, and toilet within household)

Outcome variable	Nearest-neighbour Matching		Inverse Probability Weighting		Coarsened Exact Matching	
	1993-94	2004-05	1993-94	2004-05	1993-94	2004-05
Absent from work-days per adult (15-64 years) in last one year	6.76***<br>(0.23)	1.44***<br>(0.54)	6.54***<br>(0.23)	1.28***<br>(0.52)	6.91***<br>(0.16)	1.30<br>(0.47)
Workforce participation among adults (15-64 years) in last one year (%)	-3.16***<br>(0.68)	-2.23***<br>(0.71)	-2.61***<br>(0.64)	-2.16***<br>(0.66)	-2.60***<br>(0.64)	-1.79***<br>(0.67)
Workforce participation of non-sick adults (15-64 years) in last one year (%)	13.97***<br>(0.65)	4.67***<br>(0.70)	14.29***<br>(0.61)	4.69***<br>(0.65)	14.42***<br>(0.63)	5.17***<br>(0.67)
Wage-days per adult (15-64 years) of household in last one year	-1.27<br>(2.75)	-3.97**<br>(1.74)	-14.69***<br>(3.08)	-3.35**<br>(1.71)	-12.08***<br>(2.90)	-3.59**<br>(1.68)
Wage-income per adult (15-64 years) in last one year (INR)	-412.13***<br>(108.59)	-166.55<br>(104.88)	-825.22***<br>(117.17)	-161.49<br>(103.41)	-670.77***<br>(117.91)	-135.29<br>(98.98)

Per capita cropped area by household in last one year	-0.88***<br>(0.33)	0.16<br>(0.28)	-0.58**<br>(0.30)	0.09<br>(0.29)	-0.91***<br>(0.37)	-0.01<br>(0.22)
Per capita irrigated cropped area by household in last one year	-0.67***<br>(0.24)	-0.02<br>(0.15)	-0.20<br>(0.19)	0.12<br>(0.20)	-0.78***<br>(0.30)	-0.12<br>(0.18)
Sample=Treatment + Control	2805<br>(5767)	2805<br>(5684)	10277	10072	2439<br>(5518)	2439<br>(5518)

Note: Coefficients are the average treatment effect applying nearest-neighbour matching, inverse probability weighting and coarsened exact matching using the Human Development Profile of India 1993-94 and India Human Development Survey 2004-05. Standard errors are reported in parentheses below coefficient estimates. For each coefficient, statistical significant differences between the treatment and matched controls were shown at the level of 1%***, 5%** and 10%*. Income and expenditure data in Indian Rupees in 1993-94 were inflated using the World Bank's consumer price index for India to make them comparable with 2004-05.

Table SA4: Short-term and long-term employment effects on Indian rural households affected by adult illness: results from nearest-neighbour matching, inverse probability weighting and coarsened exact matching (8 co-variates dropped: sex of household head, adult household head, religion, household size, dummy of household with three or more rooms, household with electricity, and toilet within household)

**Outcome variable**	**Nearest-neighbour Matching**		**Inverse Probability Weighting**		**Coarsened Exact Matching**	
	1993-94	2004-05	1993-94	2004-05	1993-94	2004-05
Absent from work-days per adult (15-64 years) in last one year	6.76*** (0.23)	1.37*** (0.52)	6.52*** (0.23)	1.28*** (0.52)	6.77*** (0.15)	1.50*** (0.44)
Workforce participation among adults (15-64 years) in last one year (%)	-2.77*** (0.64)	-1.53** (0.67)	-2.63*** (0.63)	-2.16*** (0.66)	-2.54*** (0.60)	-1.28** (0.64)
Workforce participation of non-sick adults (15-64 years) in last one year (%)	14.36*** (0.61)	5.37*** (0.66)	14.29*** (0.60)	4.70*** (0.65)	14.56*** (0.60)	5.66*** (0.64)
Wage-days per adult (15-64 years) of household in last one year	-2.96 (2.58)	-2.39 (1.65)	-14.86*** (3.02)	-3.55** (1.70)	-12.93*** (2.84)	-2.38 (1.56)

Wage-income per adult (15-64 years) in last one year (INR)	-530.69*** (104.01)	-134.79 (101.10)	-809.60*** (115.10)	-165.69 (102.97)	-728.84*** (115.89)	-132.22 (94.48)
Per capita cropped area by household in last one year	-0.84*** (0.30)	0.02 (0.32)	-0.55* (0.30)	-0.06 (0.29)	-0.73** (0.31)	-0.25 (0.28)
Per capita irrigated cropped area by household in last one year	-0.51** (0.21)	-0.12 (0.15)	-0.20 (0.20)	0.12 (0.21)	-0.48** (0.23)	0.03 (0.19)
Sample=Treatment + Control	2805 (6879)	2805 (6769)	10277	10072	2677 (6872)	2677 (6872)

Note: Coefficients are the average treatment effect applying nearest-neighbour matching, inverse probability weighting and coarsened exact matching using the Human Development Profile of India 1993-94 and India Human Development Survey 2004-05. Standard errors are reported in parentheses below coefficient estimates. For each coefficient, statistical significant differences between the treatment and matched controls were shown at the level of 1%***, 5%** and 10%*. Income and expenditure data in Indian Rupees in 1993-94 were inflated using the World Bank's consumer price index for India to make them comparable with 2004-05.

Table SA5: Employment effects of current adult illness and lagged adult illness on Indian rural households: results from fixed-effect regression and regression, respectively

Outcome variable (1)	Impact of current illness (FE) (2)	Impact of lagged illness (regression) (3)
Absent from work-days per adult (15-64 years) in last one year	6.30*** (0.48)	1.19 (0.74)
Workforce participation among adults (15-64 years) in last one year (%)	-1.05 (0.85)	-1.78** (0.79)
Workforce participation of non-sick adults (15-64 years) in last one year (%)	7.69*** (0.85)	6.07*** (0.79)
Wage-days per adult (15-64 years) of household in last one year	-5.69* (3.46)	-1.78 (1.85)
Wage-income per adult (15-64 years) in last one year (INR)	-381.23** (189.08)	-118.04 (109.31)
Per capita cropped area by household in last one year	-1.02** (0.49)	-0.17 (0.26)
Per capita irrigated cropped area by household in last one year	-0.76 (0.47)	-0.27 (0.26)
Sample size	10169	10072

Note: Coefficients are the effects of adult illness applying fixed-effect (column 2) using panel data (1993-94, 2004-05) and regression of lagged illness (column 3) from the Human Development Profile of India 1993-94 and employment outcomes from the India Human Development Survey 2004-05. Standard errors are reported in

parentheses below coefficient estimates. For each coefficient, statistical significance were shown at the level of 1%***, 5%** and 10%*. Income and expenditure data in Indian Rupees in 1993-94 were inflated using the World Bank's consumer price index for India to make them comparable with 2004-05.
